# A longitudinal study of mental health symptoms in young prisoners: exploring the influence of personal factors and the correctional climate

**DOI:** 10.1186/s12888-016-0803-z

**Published:** 2016-04-06

**Authors:** Leonel C. Gonçalves, Jérôme Endrass, Astrid Rossegger, Anja J. E. Dirkzwager

**Affiliations:** Department of Mental Health Services, Office of Corrections, Canton of Zurich, Hohlstrasse 552, P.O. Box 8090, Zurich, Switzerland; Department of Psychology, University of Konstanz, Universitätsstrasse 10, 78464 Konstanz, Germany; Netherlands Institute for the Study of Crime and Law Enforcement, 71304, 1008 BH Amsterdam, Netherlands

**Keywords:** Mental health symptoms, Correctional climate, Young prisoners, Longitudinal research, Personal and environmental factors

## Abstract

**Background:**

Despite the high prevalence rate of mental health problems among young prisoners, little is known about the longitudinal course and covariates of their mental health symptoms during incarceration, especially the influence of the correctional climate. The current study aimed: (1) to examine changes in young prisoners’ mental health symptoms during incarceration, (2) to identify personal factors associated with their mental health symptoms and perceptions of the correctional climate, and (3) to test the incremental effect of perceptions of the correctional climate on mental health symptoms.

**Methods:**

Data were obtained from a sample of 75 youths (aged 17 to 22 years) detained in a Portuguese young offender prison. Data were gathered 1, 3, and 6 months after their admission in this facility. Socio-demographic, clinical and criminological variables were collected. Mental health symptoms and perceptions of the correctional climate were assessed through self-report assessment tools. Linear and logistic (multi-level) regressions and tests for differences between means were performed to analyze the data.

**Results:**

Overall, mental health symptoms marginally declined by the sixth month in prison. Prisoners with a history of mental health treatment were more likely to have increased symptoms. Higher levels of mental health symptoms were associated with a history of mental health treatment, remand status, and a lower educational level. Better perceptions of the correctional climate were associated with Black race and participation in prison activities. A negative perception of the correctional climate was the strongest covariate of young prisoners’ mental health symptoms and had incremental validity over that of personal variables.

**Conclusions:**

The results highlight that both characteristics of the prisoners and of the prison environment influence young prisoners’ mental health. Prison management can try to reduce young prisoners’ mental health problems by developing scientific procedures for their mental health assessment and creating a more beneficial correctional climate.

## Background

The prevalence of any mental health disorder among young prisoners is up to 70 % [1–3]. Such high prevalence rates result in substantial mental health needs and pose a number of challenges to the criminal justice system. For instance, mental health symptoms in prison have been linked to violence, self-harm, suicide, victimization [4–7], and to a reduced willingness or capacity of prisoners to participate in daily activities and prison programs, which in turn may limit their well-being and rehabilitation [8, 9]. Moreover, high levels of mental health symptoms may result in a higher use of prison healthcare services, and subsequently increase institutional costs.

It has been argued that time spent in prison may deteriorate prisoners’ mental health, especially among those with pre-existing mental illness and more severe problems [10, 11]. Accurate knowledge about the longitudinal course of prisoners’ distress during incarceration is important because it informs prison management about high-risk periods, which may enhance an optimal timing of treatment and interventions. At present, longitudinal studies on mental health problems among incarcerated adolescents are scarce, but the existing studies suggest that symptoms of anxiety and depression declined over a two month period in prison [12, 13], and that their mental health in general improved during the first six months in custody [14].

The prevalence of mental health problems and their longitudinal course may vary across different prisoners [11]. Correlational studies on mental health symptoms in young prisoners have identified several personal risk factors, like female gender, White race, socio-economic and academic/occupational deficiencies, traumatic experiences, reduced social support, coping style, substance use, and brain injuries [12, 13, 15–20], results that tend to be consistent with the literature among adult prisoners.

In addition to personal factors, prisoners’ mental health may be related to the features of the prison environment [21]. For instance, a lack of peer support, fear of victimization, negative staff-prisoner interactions and limited daily activities may impair young prisoners’ psychological adaptation [9, 15, 22]. Such findings suggest that young prisoners’ mental health may be affected by aspects of the correctional climate. Correctional climate refers to the social, emotional, organizational and physical characteristics of a prison as perceived by its members [23]. It is the set of structural properties or conditions that are assumed to exert a major influence on prisoners’ behavior [24]. In line with the transactionist theory of prisoners’ adjustment [24, 25], environmental characteristics, in interaction with individual characteristics, may act as a source of pressure for prisoners’ behaviour.

Prior research [25–27] has identified a number of prisoners’ concerns about environmental attributes. According to Toch [25], major concerns include privacy, safety, structure (environmental stability and predictability), support, emotional feedback (being loved, appreciated and cared for), social interactions, daily activities, and freedom (autonomy). Different self-report assessment tools to measure the concept of correctional climate have been developed (see [23]). Studies using such standardized tools have linked a more positive judgment of the correctional climate to positive outcomes, like treatment motivation, internal locus of control, lower levels of aggression, and less victimization [27–29].

In the present study, we argue that perceptions of the correctional climate may also be associated with mental health issues. At present, however, research linking the correctional climate to mental health symptoms is lacking. Similarly, the characteristics of youths who identify the correctional climate as more positive are not known. Moreover, prior research on predictors of prisoners’ mental health problems – like education, mental treatment history, penal status, and index offense – is mostly based on adult samples and Anglo-Saxon countries, thus limiting generalizations [11, 21, 30–32]. In addition, most studies are cross-sectional in nature. Therefore, they are unable to provide information on patterns of mental health symptoms or to identify any causal relationship.

In an attempt to fill these gaps in knowledge, the aims of the present study were threefold: (1) to examine changes in mental health symptoms among adolescent prisoners in Portugal during their first six months in a youth correctional facility; (2) to identify individual factors–socio-demographic, clinical, and criminological–associated with their mental health symptoms and perceptions of the correctional climate; and (3) to test the incremental influence of the perceived correctional climate on mental health symptoms.

## Methods

### Setting

Data for this study were collected in the only Portuguese prison exclusively housing young males aged 16 to 21 years old (extendable to 25), who come mainly from urban areas.^1^ This high security prison includes five operational units that house remand and sentenced offenders (separated), a drug-free unit, and a well-equipped health care unit. The prison also has several courtyards, gardens, vineyards, a school, a church, and several places for work activities. The prison’s capacity is 214 cells; in 2013, the occupancy rate was 98 %. Most inmates in this correctional facility are held in individual cells.

New prisoners are assessed within 24 h by a nurse, who handles any immediate needs and determines further needs and necessary services. A physician conducts a more thorough physical and mental examination of the prisoner within the first 72 h. In this facility, prisoners are also screened by a psychologist at intake. Those prisoners considered at-risk for mental health problems are engaged in further sessions for psychological/psychiatric evaluation and treatment. Other prisoners are told that they can request a psychological consultation at any time.

After assessments by the educational and clinical staff within the first 72 h, the prisoners are sent to the “Observation” unit for an initial evaluation period that guides the development of their rehabilitation plan. During this period, which lasts around 60 days, few prisoners engage in activities and most spend around 20 h a day inside their cells. Progressively, the prisoners are enrolled in work, school, and other activities and moved to other units. Those at more advanced stages of their sentence and with good institutional behavior may qualify for the open regime and may be moved to a block allowing more freedom and autonomy in preparation for their release (see also [33]).

Although some prisoners had entered the prison directly for the first time, most had been transferred from other prisons (68 %) or had served a prior prison sentence (9 %). Our initial intention was to include only new receptions as in prior research [11, 14], but this would have reduced the sample size of this study considerably. Therefore, all prisoners entering the facility were included. For prisoners who had previously served time in prison, we assumed that they were better adapted to the prison environment and, therefore, might have better perceptions of the correctional climate.

### Procedure

Data were collected during the first (*n* = 75), third (*n* = 67), and sixth month (*n* = 60) after the prisoners’ arrival at the current prison facility.^2^ All prisoners who entered the facility between March 2011 and December 2011 were invited to participate in the study. Only those who did not understand Portuguese were excluded (approximately 6 %, mostly Romanians). Newcomers were informed face-to-face about the study, its objectives, that participation was voluntary, and that data would remain confidential. All approached prisoners (the same number as those invited) initially agreed to participate. Subsequently, the prisoners filled out the questionnaires on mental health symptoms and perceptions of the correctional climate in small groups in a private room. For those who had reading problems, the researcher read the questions and recorded the chosen answers on the questionnaires. In subsequent assessments, prisoners could fill out the questionnaires in their cells and return them in a sealed envelope.

This study combines different sources of data. Information on the socio-demographic and clinical characteristics was based on prisoners’ self-reports. Criminological data and the number of visits during incarceration were retrieved from the prison’s electronic database. All data were collected by the first author.

### Participants

The sample includes 75 males aged 17 to 22 years (*M* = 19.15 years; *SD* = 1.40) at the time of their admission to the institution. Their educational level ranged between one and 12 years of schooling (*M* = 6.85, *SD* = 2.35). The sample was diverse in terms of race (45 % Black, *n* = 34) and nationality (59 % Portuguese, *n* = 44). The majority had a history of drug use (80 %, *n* = 60); 37 % (*n* = 28) had received mental health treatment prior to their arrival in the facility. Only 40 % (*n* = 30) had already been sentenced at the time of their admission (57 % after 6 months, *n* = 34). Most prisoners were accused of property crimes (71 %, *n* = 53). The others were detained for violent (16 %, *n* = 12) and drug-related offenses (13 %, *n* = 10). Their time spent in prison before entering this institution (for the present and/or past sentences) spanned an average of 7.45 months (*SD* = 9.31, range 0–42).

### Measures

*Mental health symptoms.* At each assessment wave, mental health problems were assessed with the Portuguese version of the Brief Symptom Inventory (BSI; [34, 35]).^3^ The BSI consists of 53 mental health symptoms, and participants rate on a five point scale the extent to which they had experienced each symptom in the past week (0 = not at all, 4 = extremely). Symptoms are related to nine subscales: Somatization, Obsessive-Compulsive, Interpersonal Sensitivity, Depression, Anxiety, Hostility, Phobic Anxiety, Paranoid Ideation, and Psychoticism. The instrument also provides a total score indicating the overall level of psychological distress (i.e., the Global Severity Index; GSI). In the current study, the Cronbach’s alpha of the GSI was very high in the three waves (α_1_ = .94, α_2_ = .94, α_3_ = .95).

*Perceptions of the correctional climate.* Perceptions of the correctional climate was measured with the short version of the Prison Environment Inventory (PEI; [24]), which was translated and adapted to Portuguese for the purpose of this study.^4^The instrument includes 48 items in which prisoners indicate on a four point scale the extent to which they agreed with statements about the prison environment (0 = never, 3 = always). The items are based on Toch’s [25] eight environmental concerns: Privacy, Safety, Structure, Support, Emotional Feedback, Social Stimulation, Activity and Freedom. Because the instrument is still not validated for the Portuguese population, only the total score was used in the present study. A higher score indicates a more positive judgment of the correctional climate. The Cronbach’s alpha was good in the three waves (α_1_ = .74, α_2_ = .83, α_3_ = .81). The mean total score was 84.08 (*SD* = 13.36), 85.80 (*SD* = 13.68), and 87.54 (*SD* = 14.96) at wave one, two, and three, respectively, and there were no significant differences between waves.

*Other covariates.* Besides the number of months spent in the current facility (measured as a categorical or continuous variable, depending on the analysis), several socio-demographic, clinical, and criminological characteristics of the inmates were investigated. These characteristics included: age (continuous), years of completed education (continuous), marital status (0 = married/living together, 1 = single), nationality (0 = foreigner, 1 = Portuguese), race (0 = White, 1 = Black), the number of visits received in prison (count), drug use (in the present and/or past; 0 = no, 1 = yes), mental treatment history (0 = no, 1 = yes), index offense (categorical; drug, property, or violent), penal status (0 = remand, 1 = sentenced), participation in prison activities (work and/or school; 0 = no, 1 = yes), and time previously served in correctional facilities (continuous).

### Analyses

First, to examine overall changes in mental health symptoms during incarceration, pooled linear regression analyses were performed, regressing each BSI subscale and total score (GSI) on time in prison (categorically coded: 1, 3, and 6).^5^ To correct for the correlation in individual errors, standard errors were clustered by prisoners. When the omnibus Wald *χ*^2^ was statistically significant, contrasts of marginal linear predictions with Bonferroni correction were computed to identify significant pairwise differences between assessment waves (Table 1). Because there may be prisoners with increased or decreased symptom load, which may have been hidden in the overall change over time,^6^ we calculated the proportion of prisoners showing increases or decreases on each BSI subscale and total score from the first to the third months and from the third to the sixth months. A one-sample test of proportions was also calculated to ascertain if there was a significant difference between increases and decreases (Table 2). In addition, to identify which prisoners were associated with increases in mental health symptoms over time in prison, logistic regression was used to predict changes (dichotomous variable, 0 = decrease, 1 = increase) in total mental health symptoms depending on the independent variables of this study (Table 3).

**Table 1:** Level and Changes in Mental Health Symptoms over Time in Prison

	Month 1 (*n* = 70)	Month 3 (*n* = 58)	Month 6 (*n* = 49)	
BSI Subscale	Mean (*SD*)	Mean (*SD*)	Mean (*SD*)	Mean difference/Contrast
Somatization^a^	0.76 (0.82)	0.75 (0.74)	0.62 (0.70)	*χ* ^2^ = 6.70*
				M6 vs. M3*
Obsessive-compulsive	1.21 (0.70)	1.26 (0.81)	1.06 (0.78)	*χ* ^2^ = 6.97*
				M6 vs. M3*
Interpersonal sensitivity^a^	1.04 (0.83)	1.09 (0.77)	0.97 (0.86)	*ns*
Depression	1.28 (0.78)	1.26 (0.86)	1.12 (0.83)	*ns*
Anxiety^a^	0.93 (0.79)	0.86 (0.72)	0.77 (0.71)	*ns*
Hostility^a^	0.96 (0.69)	0.99 (0.89)	0.93 (0.74)	*ns*
Phobic anxiety^a^	0.68 (0.67)	0.63 (0.67)	0.52 (0.65)	*χ* ^2^ = 5.88^†^
				M6 vs. M1^†^
Paranoid ideation^a^	1.25 (0.69)	1.34 (0.88)	1.31 (0.85)	*ns*
Psychoticism^a^	1.09 (0.78)	1.10 (0.77)	1.07 (0.89)	*ns*
GSI^a^	1.04 (0.63)	1.03 (0.65)	0.93 (0.65)	*χ* ^2^ = 5.87^†^
				M6 vs. M3^†^

*Note. SD* Standard deviation, *M1* Month 1, *M3* Month 3, *M6* Month 6, *ns* not significant, *BSI* Brief Symptom Inventory, *GSI* Global Severity Index

^a^ Distribution normalized through square root transformation. Descriptive statistics are based on the original data. Mean differences analyses are based on the transformed variables

^†^ *p* < .10, * *p* < .05, two-tailed

**Table 2:** Proportion of Prisoners with Increased or Decreased Mental Health Symptoms over the Third- and Sixth-Month Time Periods

	From Month 1 to Month 3 (*n* = 54)			From Month 3 to Month 6 (*n* = 43)		
BSI Subscale	% increase	% decrease	Proportion test (*z*)	% increase	% decrease	Proportion test (*z*)
Somatization	46	35	1.69^†^	26	42	−2.12*
Obsessive-Compulsive	46	43	0.45	30	56	−3.43***
Interpersonal Sensitivity	55	30	4.01***	23	56	−4.36***
Depression	41	37	0.61	42	47	−0.66
Anxiety	46	43	0.45	44	42	0.27
Hostility	44	44	0.00	44	33	1.53
Phobic Anxiety	31	37	−0.91	23	47	−3.15**
Paranoid Ideation	56	39	2.56*	40	44	−0.53
Psychoticism	52	37	2.28*	40	44	−0.53
GSI	52	48	0.59	33	63	−4.07***

*Note. z* test of statistical significance, *GSI* Global Severity Index

^†^ *p* < .10, * *p* < .05, ** *p* < .01, *** *p* < .001, two-tailed

**Table 3:** Covariates of Increases and Decreases in Total Mental Health Symptoms (GSI) over the Third- and Sixth-Month Time Periods

	From Month 1 to Month 3 (*n* = 54)			From Month 3 to Month 6 (*n* = 43)		
Variable	*OR*	95 % CI	*z*	*OR*	95 % CI	*z*
Age	1.01	[0.67, 1.52]	0.05	1.09	[0.69, 1.73]	0.37
Education	0.96	[0.77, 1.20]	0.37	0.88	[0.67, 1.15]	0.95
Single	1.43	[0.34, 6.03]	0.49	2.95	[0.31, 28.14]	0.94
Portuguese	0.98	[0.33, 2.88]	0.04	1.23	[0.34, 4.54]	0.32
Black	0.88	[0.30, 2.62]	0.23	0.94	[0.25, 3.45]	0.10
Visits	1.02	[0.94,1.10]	0.43	1.00	[0.92, 1.08]	0.94
Drug use	2.50	[0.55, 11.27]	1.19	2.26	[0.23, 22.42]	0.70
Mental treatment	0.76	[0.25, 2.27]	0.50	3.50	[0.88, 13.99]	1.77^†^
Offense: drug	0.40	[0.09, 1.80]	1.19	1.33	[0.20, 9.08]	0.29
Offense: property	1.12	[0.36, 3.47]	0.19	0.51	[0.12, 2.13]	0.92
Offense: violent	2.09	[0.46, 9.41]	0.96	2.18	[0.38, 12.58]	0.87
Sentenced	1.36	[0.47, 3.99]	0.57	1.66	[0.45, 6.13]	0.77
Prison activity	0.96	[0.77, 1.20]	0.37	1.66	[0.45, 6.13]	0.77
Prior time served	1.02	[0.97, 1.08]	0.80	1.03	[0.96, 1.10]	0.85
PEI	1.00	[0.96, 1.04]	0.17	1.01	[0.97, 1.06]	0.75

*Note. OR* odds ratio, *CI* confidence interval, *z* test of statistical significance, *GSI* Global Severity Index, *PEI* Prison Environment Inventory

^†^ *p* < .10, two-tailed

Second, to identify personal factors associated with mental health symptoms and perceptions of the correctional climate, initially, bivariate linear multi-level regressions were conducted, regressing the BSI and PEI total scores on the independent variables (Table 4).^7^ Random-effect models with robust standard errors were utilized to take into account variations across individuals and to analyze time-invariant variables. Then, variables that were significant in the bivariate analyses were included in a multiple regression model. Since the sample size of the current study was limited, variables were removed from the multiple regression models one by one by order of (low) statistical significance until only those significant at the 5 % level remained (time in prison was included anyway).

**Table 4:** Covariates of Mental Health Symptoms and Perceptions of the Correctional Climate

	Mental Health Symptoms^a^			Correctional Climate^b^		
Variable	*b*	95 % CI	*z*	*b*	95 % CI	*z*
Time in prison	−0.007	[−0.019, 0.004]	1.28	0.028	[−0.017, 0.073]	1.22
Age	−0.001	[−0.046, 0.047]	0.03	−0.055	[−0.182, 0.073]	0.84
Education	−0.111	[−0.167, −0.055]	3.89***	0.086	[−0.126, 0.297]	0.79
Single	−0.012	[−0.194, 0.171]	0.13	−0.105	[−0.725, 0.515]	0.37
Portuguese	0.187	[0.043, 0.330]	2.55*	−0.228	[−0.632, 0.176]	1.11
Black	−0.288	[−0.421, −0.155]	4.23***	0.553	[0.155, 0.951]	2.73**
Visits	0.008	[0.002, 0.015]	2.60**	−0.011	[−0.031, 0.009]	1.09
Drug use	0.112	[−0.078, 0.301]	1.16	−0.401	[−0.913, 0.110]	1.54
Mental treatment	0.309	[0.165, 0.453]	4.21***	−0.264	[−0.657, 0.129]	1.32
Offense: drug	−0.098	[−0.295, 0.098]	0.98	−0.143	[−0.744, 0.458]	0.47
Offense: property	0.063	[−0.098, 0.224]	0.77	−0.148	[−0.628, 0.331]	0.61
Offense: violent	−0.012	[−0.223, 0.200]	0.11	0.355	[−0.279, 0.989]	1.10
Sentenced	−0.231	[−0.361, −0.101]	3.48**	0.348	[0.051, 0.747]	1.71^†^
Prison activity	−0.043	[−0.098, 0.012]	1.55	0.270	[0.032, 0.508]	2.22*
Prior time served	−0.013	[−0.021, −0.005]	3.03**	0.019	[−0.001, 0.038]	1.87^†^
PEI	−0.104	[−0.139, −0.069]	5.76***	-	-	-
GSI	-	-	-	−1.180	[−1.649, −0.710]	4.93***

*Note. b* unstandardized regression coefficient, *CI* confidence interval, *z* test of statistical significance, *GSI* Global Severity Index, *PEI* Prison Environment Inventory

^a^ *n* = 74, observations = 176

^b^ *n* = 74, observations = 182

^†^ *p* < .10, * *p* < .05, ** *p* < .01, *** *p* < .001, two-tailed

Third, to test the incremental effect of perceptions of the correctional climate on mental health symptoms, the PEI total score was added to the variables of the multiple regression model for predicting mental health symptoms. Multi-level coefficients of determination (*R*^2^) were calculated using the formula exposed in Rabe-Hesketh and Skrondal ([36], p.102) in order to examine the increase in the explained variance after including perception of the correctional climate in the model (Table 5). There was no multicollinearity between variables (variance inflation factors < 1.12) and the model was well-specified (hat^2^*z* = 1.27, *ns*). Time in prison was centered at month 1. The remaining continuous variables were grand-mean centered.

**Table 5:** Incremental Effect of Perceptions of the Correctional Climate on Mental Health Symptoms (GSI)

	Model 1			Model 2		
Variable	*b*	[95 % CI]	*z*	*b*	[95 % CI]	*z*
Intercept	−0.013	[−0.070, 0.095]	0.30	−0.006	[−0.065, 0.077]	0.16
Time	−0.004	[−0.015, 0.007]	0.72	−0.002	[−0.013, 0.008]	0.40
Mental treatment	0.236	[0.109, 0.364]	3.63***	0.215	[0.099, 0.331]	3.63***
Sentenced	−0.191	[−0.310, −0.072]	3.15**	−0.171	[−0.273, −0.070]	3.31**
Education	−0.065	[−0.113, −0.017]	2.63**	−0.060	[−0.104, −0.016]	2.65**
PEI	-	-	-	−0.094	[−0.127, −0.062]	5.75***
RE variance	0.053***			0.046***		
Wald *χ* ^2^	43.53***			102.86***		
*R* ^2^	.360			.439		
Δ*R* ^2^	-			.079		

*Note. n* = 74, observations = 176; *b* unstandardized regression coefficient; *CI* 95% confidence interval; *z* test of statistical significance; *RE* random-effect (intercept); *Wald χ* ^2^ omnibus test of model significance, *R* ^2^ coefficient of determination, Δ*R* ^2^ improvement in *R* ^2^, *GSI* Global Severity Index, *PEI* Prison Environment Inventory

** *p* < .01, *** *p* < .001, two-tailed

One participant was removed from all analyses for being an outlier (*se*_i_ > 2.50), resulting in a final sample of 74 prisoners. BSI scales that were not normally distributed based on Shapiro-Wilk tests of normality were normalized with a square root transformation for inferential analyses. Data missing at random in BSI and PEI items were imputed manually based on the predicted probabilities of regression models.^8^ Questionnaires with 25 % or more of missing data were excluded (5 %, *n* = 4 on the BSI at wave 1). The analyses were conducted in the software Stata 13 for windows 7.

### Ethical approval

The protocol of this study was approved by the ethic committee of the Portuguese General Directorate of Reintegration and Prisons, Ministry of Justice. All prisoners signed an informed consent form for participation and publication of clinical data.

## Results

### Patterns of mental health symptoms

The level and changes in young prisoners’ mental health symptoms during their first six months in the current prison facility are presented in Table 1. The total level of mental health symptoms (GSI) remained stable between the first and the third month, and then decreased at the sixth month. The Wald test confirmed that the average level of mental health symptoms marginally changed over time (*χ*^2^ (2) = 5.87, *p* = .053), and the contrast test revealed that mental symptoms were marginally lower in the sixth month compared to the third month (*p* = .077). Similarly, the level of somatization and the level of obsessive-compulsive symptoms was significantly lower in the sixth month than in the third month (*p* = .031; *p* = .030, respectively). In addition, symptoms of phobic anxiety were marginally lower at the sixth month than in the first month (*p* = .075).

The proportion of prisoners with increased or decreased mental health symptoms are presented in Table 2. From the first to the third month, more prisoners had an increased rather than decreased symptom load, unless the hostility and phobic anxiety subscales were included. Proportion tests showed that increases during this time period were significantly higher than decreases on the somatization (*p* = .090), interpersonal sensitivity (*p* > .001), paranoid ideation (*p* = .010), and psychoticism (*p* = .022) subscales. On the contrary, more prisoners had a decreased symptom load from the third to the sixth month, unless symptoms were related to anxiety and hostility. The proportion of prisoners with decreases was significantly higher than those with increases on the somatization (*p* = .033), obsessive-compulsive (*p* > .001), interpersonal sensitivity (*p* > .001), and phobic anxiety subscales (*p* = .002), as well as on the GSI (*p* > .001).

Table 3 presents the logistic regression analyses predicting increases in mental health symptoms over specific time periods. No variable was significantly associated with increases in mental health symptoms from the first to the third month. However, from the third to the sixth month, against the general tendency for decreased mental health symptoms, prisoners with a history of mental health treatment were marginally associated with increased mental health symptoms. Specifically, prisoners with a mental health treatment history were 3.5 times more likely (*z* = 1.77, *p* = .076) to have an increased symptom load from the third to the sixth month compared to prisoners without a history of mental health treatment.

### Covariates of mental health symptoms and perceptions of the correctional climate

The bivariate regression analyses of mental health symptoms (GSI) and perceptions of the correctional climate on the independent variables are presented in Table 4.

Having a Portuguese background, receiving more visits in prison, and a history of mental health treatment were significantly associated with higher levels of mental symptoms. Contrary, Black prisoners, prisoners with a higher educational level, sentenced prisoners, and prisoners who served more time in prison prior to their arrival in the current institution, reported lower levels of mental health problems. Moreover, prisoners who judged more positively about the correctional climate also reported lower levels of mental health symptoms. Personal factors that were significant predictors of mental health symptoms in the bivariate analyses were then entered into a multiple regression model. Three personal factors remained significantly associated with the outcome. Young prisoners with a history of mental health treatment (*z* = 3.63, *p* < .001), who had not been sentenced yet (*z* = −3.15, *p* = .002), and who had a lower educational level (*z* = −2.63, *p* = .009) reported a higher level of mental health symptoms. These results are presented in Model 1 of Table 5.

On the other hand, Black prisoners and prisoners who participated in prison activities had better perceptions of the correctional climate, as did sentenced prisoners and prisoners having served a longer prior time in prison, although these two exploratory variables were only marginally significant. When entered together into a multiple regression analysis, Black race (*z* = 2.64, *p* = .008), and participation in prison activities (*z* = 2.03, *p* = .042) remained significant and accounted for 10 % of the variance in young prisoners’ perceptions of the correctional climate. The effect of time was not significant, but variation across prisoners in their perceptions of the correctional climate was substantial (RE = 0.555, *p* < .001).

### Incremental effect of perceptions of the correctional climate on mental health symptoms

In Model 2 of Table 5, we added perceptions of the correctional climate to the model predicting mental health symptoms to test the incremental effect of this variable. Having a history of mental treatment, remand status, and a lower educational level remained significant covariates of mental health symptoms (*z* = 3.63, *p* < .001; −3.31, .001; and −2.65, .008 respectively). Perceptions of the correctional climate turned out to be the most significant covariate in the model. Young prisoners who judged more positively about the correctional climate in the facility reported fewer mental health symptoms (*z* = −5.75, *p* < .001). After adding this variable, the statistical significance of the model (*χ*^2^ (5) = 102.86 vs. (4) 43.53) and the proportion of explained variance increased substantially (*R*^2^ = .439 vs. .360). Perceived correctional climate accounted for almost 8 % of the variance in mental health symptoms during incarceration after controlling for personal factors.

In the multi-level analyses, time in prison was not a significant predictor of mental health. We tested the random-effect of time in prison through a mixed-effect model but it was not a better fit to the data.^9^ This means that the effect of time in prison disappeared when accounting for differences across prisoners, and the patterns of mental health symptoms in our sample considerably varied in initial level (RE = 0.046, *p* < .001) but not in shape.

## Discussion

The current study examined the longitudinal course of mental health symptoms among a sample of young prisoners in Portugal and attempted to identify covariates of their mental health. The study contributes to current knowledge because longitudinal studies on the development and covariates of mental health symptoms during imprisonment are rare, particularly among young prisoners. Furthermore, studies investigating covariates of perceptions of the correctional climate and the influence of this construct on young prisoners’ mental health were lacking, although it is known that environmental factors can influence the way inmates adjust to prison life.

The present study showed that young prisoners reported elevated levels of mental health symptoms. Compared with non-self injuring adolescent students in Portugal [37], our sample showed higher levels of mental health symptoms with respect to all subscales and the total BSI score across the three time periods of assessment. This finding shows that youths in the correctional justice system have higher mental health needs than those in the general population. Similarly, our sample reported higher levels of mental health symptoms in all of the BSI subscales across the three time periods of assessment compared to those found in adult male prisoners in Portugal (except anxiety, which was only higher at the first month) [38]. This is in line with prior research highlighting that women, juveniles, and older prisoners have increased healthcare needs [39].

In accordance with the few previous longitudinal studies [12–14], we observed that mental health symptoms among young prisoners’ in Portugal tend to decline during their first six months in the prison facility, but only after the third month. During the first three months, the levels of symptoms were augmented in several BSI subscales for a significant proportion of prisoners, which may be associated with the initial shock of imprisonment, either for those directly entering this establishment or those who were transferred to this new correctional prison facility. After this period, factors like free access to healthcare, reduced opportunities to use drug and alcohol, the daily structure of imprisonment, and the process of adjustment to prison life, may potentially explain reductions in prisoners’ mental symptoms during imprisonment [11, 39]. However, when accounting for the unique attributes of the prisoners, time was no longer associated with overall reductions in mental health symptoms. Also, patterns of mental health symptoms across prisoners were significantly varied in their initial level but not in shape. Therefore, prison mental healthcare services may not be very effective in identifying and addressing the mental health problems of the youths with higher treatment needs. This observation was further supported by the fact that the only group of prisoners associated with increases in overall mental health symptoms after the third month was prisoners with a history of mental health treatment, showing that young prisoners with prior vulnerabilities were those more likely to worsen during their time in prison [10, 11].

A history of mental health treatment, remand status, and lower educational level were robust personal risk factors for young prisoners’ mental health symptoms. These results confirm findings among adult prison populations [11, 21, 30–32] and, therefore, suggest that these risk factors can be generalized to offenders in youth correctional facilities. Prisoners with a history of mental health treatment may have higher pre-existing vulnerabilities and may continue to have higher mental healthcare needs upon entry in prison. Also, being on remand may be a source of additional stress due to the uncertainty regarding the future and the often more restrictive prison conditions in remand custody [11]. In addition, it is possible that prisoners with a lower educational level may possess fewer cognitive and social skills to deal with the challenges of prison life [31].

The strongest predictor of young prisoners’ mental health was, however, the way they perceived the correctional climate of the facility. Prisoners who felt more positive about the correctional climate reported lower levels of mental health symptoms during their incarceration. This confirms the importance of environmental factors and shows that environmental factors do not only shape prisoners’ behavior [24, 25] but also their well-being in prison, thus extending the transactionist theory of prisoners’ adjustment. In the present study, we could only examine the effect of the overall opinion about the correctional climate on young prisoners’ mental health. An interesting avenue for future research would be to validate the PEI for the Portuguese population and examine in more detail which specific aspects of the correctional climate are particularly related to prisoners’ mental health.

Finally, this study showed that prisoners who are Black and participate in prison activities tend to have better perceptions of the correctional climate. Prior research showed that juvenile Black prisoners generally reside under more adverse environmental conditions, reporting higher levels of delinquency, violence, victimization, gang fighting, and witnessing severe injury and death [19]. Such background characteristics, combined with the large proportion of Black prisoners in the current institution, which may increase their solidarity and influence, could make them better prepared to face the hardship of imprisonment and shape their perceptions of the correctional climate in a more positive way. In addition, previous research demonstrates that young prisoners who participated in prison activities were associated positively with autonomy and well-being and negatively with aggressive behavior. Furthermore, participation in prison activities reduced boredom, decreased anxiety, and brought a positive atmosphere to the group [9], which may also explain better perceptions of the correctional climate. If participating in prison activities influences young prisoners’ perceptions of the correctional climate, and perceptions of the correctional climate are related with mental health symptoms, then the lack of activities and long periods of isolation during the first 60 days in prison may also indirectly contribute to the higher level of mental health symptoms observed during the first three months.

However, we are not aware of prior research correlating race and participation in prison activities with perceptions of the correctional climate that supports these hypotheses. In addition, the significant variation in perceptions of the correctional climate across the prisoner population and reduced number of predictors for this outcome found in the present study underscore the need for more research to identify, explain, and address the environmental needs of prisoners at the individual level.

### Limitations and Implications

This study has some limitations. First, and perhaps of foremost importance, despite the prospective design, it remains unclear whether perceptions of the correctional climate are a cause or a consequence of mental health symptoms. It is likely that prisoners with more anxiety, depression, hostility, and other symptoms would rate the prison environment negatively. However, the covariates of perceptions of the correctional climate and mental health symptoms in multiple regression analyses were different, which shows that these two outcomes are associated with different factors. Second, the sample size was small and there was some attrition in the study, which impacted the power of the analyses and may have introduced bias. Third, this study focuses on male adolescents from only one prison in Portugal, which may limit the generalization of the findings. Replication of our findings using data from other correctional populations, settings, and countries is needed. Fourth, data on mental health problems were based on prisoners’ self-reports only and thus may suffer from single source bias and social desirability. Finally, measuring perceptions of the correctional climate is a complex issue, and the PEI was adapted and used with a Portuguese sample for the first time. Therefore, little can be inferred about the validity of this tool in our population.

Despite these limitations, this study has several implications for correctional policy and practice. First, young prisoners appear to experience high levels of mental health symptoms. Therefore, they may require more prison healthcare resources and age-specific interventions than their adult counterparts [39]. Second, the first three months in prison may represent a period of increased distress and risk among the youths. Correctional staff should therefore pay particular attention to young prisoners’ emotional state and behaviour during this period. Prisoners with a history of mental health treatment may require special observation beyond this period because they are more likely to deteriorate during imprisonment. Third, this study suggests the need of a standardized mental health screening at intake, which is still not common practice in Portugal. This screening should use a standardized instrument and should result in a more in depth clinical assessment and psychological support in prisoners found to have higher mental healthcare needs [1, 39].

In addition to the results of the screening tool, mental treatment history, remand status, and lower educational level are personal risk factors that may be used to signal young prisoners with increased mental healthcare needs and assign them to appropriate interventions and programs. This could include early mental healthcare for prisoners with a history of mental health treatment, education and coping skills training for those with lower educational levels, and specific programs and activities for the remanded prisoners.

Importantly, the current study suggests that prison authorities can try to improve young prisoners’ mental health by creating prison environments that address aspects of a positive correctional climate (e.g., privacy, security, structure, social stimulation, activity [25]). As it is difficult to modify prisoners’ pre-existing characteristics, creating prison environments that meet prisoners’ needs and promote their well-being may be a more effective way to enhance their rehabilitation [21, 23, 27]. This is especially important considering that mental health disorders have been associated with future prison spells [8].

Finally, because prisoners who participate in prison activities tend to have better perceptions of the correctional climate, which in turn appears to be associated with better mental health outcomes, more prisoners should be enrolled in prison activities starting as soon as possible upon entering prison. Although an observation period is necessary to establish a rehabilitation plan, spending about 20 h a day alone inside the cell during the first months of incarceration may be detrimental to prisoners’ mental health and is against the rehabilitative ideology of imprisonment.

## Conclusions

In conclusion, the present study enhances knowledge regarding the assessment and management of mental health symptoms among young prisoners, which are a population of increased risk and needs. The results highlight that both characteristics of the prisoners and of the prison environment influence young prisoners’ mental health. Prison management can try to reduce young prisoners’ mental health problems by developing scientific procedures for their mental health assessment and creating a more beneficial correctional climate, which may improve prisoners’ well-being and potentially reduce recidivism in crime. However, more research on personal and environmental risk factors is needed to further develop the current knowledge on this topic and assess the generalizability of these findings.

### Availability of Data and Materials

Under permission granted from the Portuguese Ministry of Justice to conduct this research, the authors are not allowed to share the dataset in order to preserve the confidentiality of the data and the persons who participated in this study.

## Abbreviations

BSI: Brief Symptom Inventory
GSI: general severity index
PEI: Prison Environment Inventory

## Footnotes

1 In Portugal, criminal responsibility starts at the age of 16.

2 The prisoners that did not participated in wave 2 and wave 3 (20 %, *n* = 15) had been released or transferred to another facility. Also, among the prisoners who remained in prison, some data on the BSI and PEI are missing at wave 2 and wave 3 (12 %, *n* = 8 and 17 %, *n* = 10, respectively) because they were serving disciplinary measures, they were in diligences in outside facilities, or simply because they did not want to participate. To evaluate the influence of attrition bias we run bivariate logistic regressions predicting attritions through the independent variables of this study. Prisoners who drop-out from the study were more likely to have a shorter prior time served in prison (*z* = −2.36, *p* = .018). With regard to the instruments, no variable was significant at the 5 % level, neither at wave 2 nor wave 3.

3 The BSI is not designed for prison populations and some items require slight adaptation to make sense in this context. Minor modifications were made in two items related to Phobic Anxiety, but respecting the underlying symptom being explored.

4 The translated PEI was previously administered to a random sample of 10 prisoners to test its face validity. Based on their comments, two items were substituted by others from the same dimension included in the longer PEI version (see [24]).

5 A pooled linear regression analyses with clustered standard errors was used instead of an ANOVA or a random-effect model because, in our data, this method provided predicted values closer to the observed values than the other two methods.

6 We thank the reviewer for this pertinent comment.

7 These analyses were performed on the GSI scale only because regressing all BSI subscales on the independent variables would have generated an extensive list of results, and the findings would not be much different. Including the predictors of Model 2 in Table 5 in multiple regression analyses, the direction of the effects was the same across all BSI subscales and all models were significant (*p* < .001). However, educational level was not significantly related with the Obsessive-Compulsive, Depression, Hostility, Paranoid Ideation, and Psychoticism subscales. Sentenced status was not significantly related with Hostility.

8 We developed an ordered logit regression model for each missing item, using the missing item as an outcome and the other items of the BSI or PEI as predictors. After fitting the best model, marginal effects were calculated to predict the prisoner’s score on the missing item based on the score of the items included in the model.

9 Likelihood-ratio tests were used to compare the fit of the pooled regression model with the random-effect model (random-effect of the intercept – initial level), and to compare the random-effect model with the mixed-model (random-effect of time). Because the mixed model did not prove to be a better fit to the data (i.e., patterns of mental health symptoms did not significantly vary over time across prisoners), the simpler random-effect model was used.
